# Cannabinoid Receptors Signaling in the Development, Epigenetics, and Tumours of Male Germ Cells

**DOI:** 10.3390/ijms21010025

**Published:** 2019-12-18

**Authors:** Marco Barchi, Elisa Innocenzi, Teresa Giannattasio, Susanna Dolci, Pellegrino Rossi, Paola Grimaldi

**Affiliations:** Department of Biomedicine and Prevention, University of Rome “Tor Vergata”, 00133 Rome, Italy; marco.barchi@uniroma2.it (M.B.); elisa.inno92@gmail.com (E.I.); teresagiannattasio20@gmail.com (T.G.); dolci@uniroma2.it (S.D.); pellegrino.rossi@med.uniroma2.it (P.R.)

**Keywords:** male germ cells, spermatogenesis, sperm, testicular tumors, embryonal carcinoma, cannabis, epigenetic, endocannabinoid system, intergenerational

## Abstract

Endocannabinoids are natural lipid molecules whose levels are regulated by specific biosynthetic and degradative enzymes. They bind to and activate two main cannabinoid receptors type 1 (CB_1_) and type 2 (CB_2_), and together with their metabolizing enzymes form the “endocannabinoid system” (ECS). In the last years, the relevance of endocannabinoids (eCBs) as critical modulators in various aspects of male reproduction has been pointed out. Mammalian male germ cells, from mitotic to haploid stage, have a complete ECS which is modulated during spermatogenesis. Compelling evidence indicate that in the testis an appropriate “eCBs tone”, associated to a balanced CB receptors signaling, is critical for spermatogenesis and for the formation of mature and fertilizing spermatozoa. Any alteration of this system negatively affects male reproduction, from germ cell differentiation to sperm functions, and might have also an impact on testicular tumours. Indeed, most of testicular tumours develop during early germ-cell development in which a maturation arrest is thought to be the first key event leading to malignant transformation. Considering the ever-growing number and complexity of the data on ECS, this review focuses on the role of cannabinoid receptors CB_1_ and CB_2_ signaling in male germ cells development from gonocyte up to mature spermatozoa and in the induction of epigenetic alterations in these cells which might be transmitted to the progeny. Furthermore, we present new evidence on their relevance in testicular cancer.

## 1. Introduction

Spermatogenesis is a highly coordinated process in which male germ cells differentiate passing through three major phases: mitotic proliferation of spermatogonia, meiosis in spermatocytes, and spermiogenesis, i.e., the morphogenetic process by which haploid round spermatids form mature spermatozoa [1]. The correct progression of germ cell differentiation and the release of a normal number of spermatozoa with fertilizing activity is regulated by gonadotropins follicle stimulating hormone (FSH) and luteinizing hormone (LH), steroid hormones, and a complex network of autocrine and paracrine factors including endocannabinoids (eCBs) [2]. The biological actions of eCBs are mediated by two main cannabinoid receptors: CB_1_ and CB_2_. Altogether, cannabinoid receptors, eCBs, and all the enzymes involved in their biosynthesis and degradation form the endocannabinoid system (ECS). ECS is deeply involved in the control of male reproduction and alterations in the system have adverse effects on various stages of germ cell differentiation, from gametogenesis to sperm motility, capacitation, and fertilizing ability [2]. In this review we summarize the most important results of cannabinoid receptors signaling in male germ cells as well as in testicular tumours.

## 2. Male Germ Cell Development

In mice, germ cell development begins with the specification of primordial germ cells (PGCs), the precursors of gametes. They are first detectable in extra-embryonic regions during the early embryonic period [3] and then migrate into the undifferentiated gonads. During migration and colonization of the gonads, PGCs are characterized by intense mitotic proliferation. In the fetal testis, PGCs differentiate into pre-spermatogonia which undergo mitotic arrest in the G1/G0 stage until birth, when quiescent cells re-starts to proliferate. At puberty, they enter the first wave of spermatogenesis characterized by spermatogonial entry into meiosis and further differentiation to form immature sperm. This process extends throughout the male adult life and comprises continuous spermatogenic cycles starting from spermatogonia stem cells. In the adult mouse testis, in which the cycle and the wave of the seminiferous epithelium are well established, undifferentiated spermatogonial stem cells (known as A-single spermatogonia (As)) can both renew themselves or produce more differentiated A-paired spermatogonia (Apr). The Apr cells then divide into A-aligned spermatogonia (Aal) that further differentiate into A1 spermatogonia [4]. The appearance of A1 spermatogonia coincides with the re-expression of the *c-Kit* gene, which encodes the receptor for the Kit ligand (KL) and which had been previously downregulated in the male fetal testis at the time of mitotic quiescence. Kit is a tyrosine kinase receptor that mediates proliferation/survival signals in type A spermatogonia [5]. Upon Kit expression, spermatogonia become sensitive to KL produced by Sertoli cells [6] and undergo a finite number of proliferative cycles (forming the A2–A4, intermediate, and B spermatogonia), before entering meiosis. Thus, the temporal appearance of Kit expression and KL sensitivity in spermatogonia marks the switch from the Aal spermatogonia to the A1–B differentiating cell types. In addition to KL, proliferation and differentiation of mitotic spermatogonia in the pre-pubertal testis, as well as the subsequent progression of the differentiation program, also depend on the production of at least two additional growth factors by the nursing Sertoli cells within the seminiferous tubules: glial cell line-derived neurotrophic factor (GDNF), acting on the spermatogonial stem cells [7], and Bone Morphogenetic Protein 4 (BMP4), which also acts directly on the spermatogonial compartment up-regulating Kit expression [8]. Following the last mitotic division, spermatogonia progresses into meiosis as preleptotene spermatocytes. The entry into meiosis is a critical step in spermatogenesis and an essential role in promoting this step is played by the all-trans retinoic acid derivative (ATRA) [9], which may control the timing of meiosis, at least partially, by influencing both the somatic and the germ cell compartment of the postnatal testis through the activation of the KL/Kit system [10] and by inducing the expression of *Stra8* gene [9,11].

In this context, we recently identified ECS as an important autocrine-paracrine regulator of meiotic entry in postnatal male germ cells [12], that acts along with ATRA in an additive manner [13]. Upon meiotic entrance, preleptotene spermatocytes undergo the last round of DNA synthesis and progress into the leptotene stage initiating the process of meiotic recombination. Recombination requires a programmed introduction into the genome of double-strand breaks (DSBs) by the topoisomerase VI-like protein SPO11 [14,15] that allows the formation of synapsis between homologous chromosomes and DNA exchanges, called crossing-overs [16]. Crossing-over formation is crucial to ensure the shuffling of the genome, as well as the proper segregation of chromosomes at the first meiotic division [16]. A key event of prophase I is the development of the synaptonemal complex (SC), a zipper-like high-order chromosome structure formed by an axial/lateral element containing the SYCP3 protein and a central element containing the protein SYCP1. The first meiotic division is then followed by a second division not preceded by DNA replication so that the two successive cell divisions produce haploid spermatids that will differentiate into sperm through a process called spermiogenesis. 

Post-meiotic stages appear to be regulated by the Sertoli cell-mediated action of androgens [17]. In the end, spermatozoa, released in the lumen of the seminipherous tubules, acquire motility during the transit in the epididymis and then capacitation within the female genital tract.

## 3. Epigenetic Modifications in Male Germ Cell Development

Several stages of male germ cell development, from PGCs specification to spermatozoa development, are regulated by epigenetic mechanisms. The establishment and maintenance of the epigenetic program during germ cell development is associated with appropriate gamete functions and alterations of the germ cell epigenome might impact fertility and, importantly, may be transmitted to the next generation [18]. 

Epigenetic modifications are heritable and possibly reversible modifications in gene expression that do not involve alterations in the DNA sequence. Epigenetic mechanisms include DNA methylation, post-translational modifications of histones, and noncoding RNA (miRNA). DNA methylation is one of the most studied epigenetic modification during development. In mammals, *de novo* DNA methylation is established by DNA methyltransferases, DNMT3A and DNMT3B, and is then maintained by DNMT1 during cell division [19]. Instead, Ten-eleven translocation methylcytosine dioxygenase (TETs) proteins, including TET1, TET2, and TET3, are crucial regulators of active DNA demethylation and catalyze the oxidation of 5-methylcytosine (5mC) to 5-hydroxymethylcytosine (5hmC) [20]. Different post-translational modifications can occur in histones such as acetylation, methylation, phosphorylation, ubiquitination, ADP addition and ribosylation, which control gene transcription.

Chromatin epigenetic reprogramming starts very early in utero in the germline, soon after primordial germ cell (PGC) specification, at around 7 days post coitum (dpc), and proceeds throughout their migration and proliferation period through erasure of DNA methylation [21] and remodeling of histone modifications [22,23]. Passive DNA demethylation along with TET1 and TET2 activation erases the epiblast-specific and the parental-specific patterns of DNA methylation, respectively, both in male and female PGCs by 13.5 dpc of development [24]. However, some methylation sites escape have been detected in germ cells [24,25], identifying potential targets loci for transgenerational inheritance. Concomitantly, histone modifications occur predominantly on H3K9me2, that starts to be inhibited at 7.25 dpc, and on H3K27me3, that increases at 8.25 dpc [26].

By 13.5 dpc up to birth, male germ cells, defined as pro-spermatogonia, initiate de novo DNA methylation by increasing DNMT3A and DNMT3L synthesis [24,27]. *De novo* DNA methylation occurs in germ cells in a sex-specific manner and CpG and non-CpG methylation are established in mitotically arrested gonocytes. In males, methylation imprints in germ cells are mostly completed before birth and are maintained post-natally. 

Another period of epigenomic reprogramming coincides with the onset of spermatogenesis and the entry into meiosis. Spermatogonia show unique histone methylation pattern such as low H3K9me2 levels [28,29] that dynamically changes following meiotic entry^12^ in parallel to a global enrichment in H3K4me3 when they reach the leptotene stage of prophase I. The trimethylation at specific H3K4, at meiotic entry, is linked to the activity of meiosis-specific KRAB-domain zinc finger methyltransferase PRDM9 [30,31,32] that specifies the recombination hotspots, where DSBs are made at leptonema, essential for progression through prophase I. Additionally, male germ cells express several histone variants, including TH2A, TH2B, TH3, H3.3A, H3.3B, and HT1, which become incorporated into differentiating spermatogonia and/or spermatocytes [33] to remodel chromatin during meiosis.

In the final stages of spermatogenesis, haploid round spermatids undergo several biochemical and morphological changes that include an extensive remodeling of chromatin in which testis-specific histones are transiently replaced first by transition proteins (TNPs; TNP1 and TNP2) and then by protamines (PRMs; PRM1 and PRM2). Protamine has a critical role in spermatid differentiation and their deficiency can lead to sperm DNA damage and embryonic death in mice [34].

## 4. Endocannabinoid System and Male Reproduction

During the last twenty years, an ever-growing number of studies have focused attention on endocannabinoids as important physiological regulators of male reproduction [2,35,36]. Endocannabinoids are natural small lipid molecules widely distributed in the body. The two most studied endocannabinoids are *N*-arachidonoylethanolamine (anandamide; AEA) and 2-arachidonoylglycerol (2-AG). AEA was the first endocannabinoid to be discovered in 1992 [37] and its name derived from the word “Ananda” which means “bliss” in Sanskrit, describing the euphoric effects of this ligand. Indeed AEA binds to and activates the same receptor as the phytocannabinoid Δ9-tetrahydrocannabinol (THC), the active principle of *Cannabis sativa* [38]. The second eCB identified was 2-arachidonylglycerol (2-AG) [39]. AEA synthesis is catalysed by an *N*-acylphosphatidylethanolamine-specific phospholipase D (NAPE-PLD) [40], while the formation of 2-AG occurs by hydrolysis of inositol phospholipids through a specific phospholipase C (PLC) to generate diacylglycerol (DAG), which is then converted into 2-AG by an sn-1-DAG lipase (DAGL) [41]. Two specific enzymes control ECs degradation: the fatty acid amide hydrolase (FAAH) that preferentially degrades AEA (2-AG to a less extent) [42] and the monoacylglycerol-lipase (MAGL) that hydrolyzes 2-AG [43]. In the brain, 2-AG is found at higher levels then AEA under most physiological conditions, indicating that 2-AG is the most efficacious endogenous natural ligand for the cannabinoid receptors [44]. AEA, but not 2-AG, also behaves as an endovanilloid, binding to and activating the type-1 vanilloid receptor (transient receptor potential vanilloid 1, TRPV1) at an intracellular site [45]. Endocannabinoids are released “on-demand” from membrane phospholipid precursors and their tone is established by the balance between their biosynthesis and degradation. The biological actions of eCBs are mediated by two main cannabinoid receptors: CB_1_ and CB_2_. Endocannabinoids and their receptors are present in the testis of invertebrates and vertebrates, including sea urchins, frogs, rats, mice, boars, and humans [35,36]. This conserved expression across species supports the fact that endocannabinoid signaling plays important roles in male reproductive functions. Strong evidence indicates that eCBs and signaling cascades via cannabinoid receptors regulate different biological activities on male reproduction by acting at central and gonadal levels [46]: they modulate pituitary-gonad (HPG) axis, Sertoli and Leydig cells functions, germ cell differentiation [2], and sperm functions [47,48]. Consequently, any changes in the tone of eCbs and/or in the expression of cannabinoid receptors CB_1_ or CB_2_ may have consequences on reproductive health. Accordingly, in humans, low AEA levels and decreased cannabinoid receptor mRNA transcripts have been found in seminal plasma of men with asthenozoospermia or oligoasthenoteratozoospermia [49,50].

**Figure 1:**
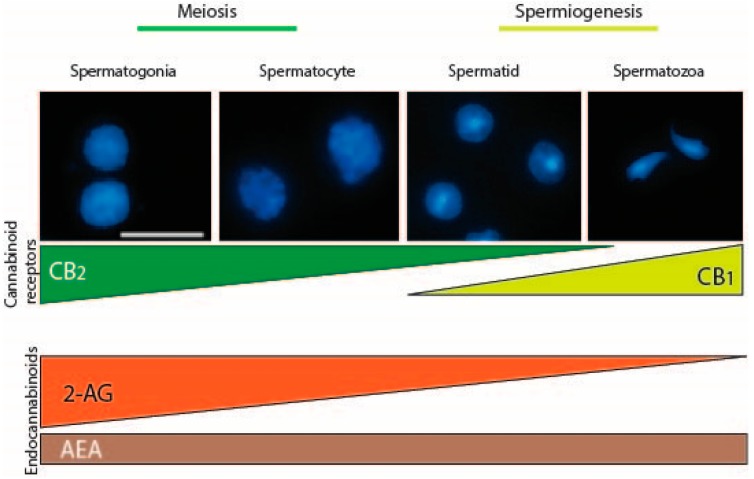
Endocannabinoid system in male germ cell development. Mammalian male germ cells at different stages of differentiation, from mitotic cells (spermatogonia), meiotic cells (spermatocytes) up to haploid stage (spermatids) and spermatozoa, have a complete and active ECS which is modulated during spermatogenesis. In the mouse, ECS controls at least two critical steps of the germ cell differentiation: meiosis and spermiogenesis. In mouse testis, spermatogonia express the higher level of CB_2_ receptor that dramatically decreases in spermatocytes, reaching a very low level in spermatids and disappearing in mouse spermatozoa. On the contrary CB_1_ starts to be expressed in haploid cells. Interestingly, spermatogonia also possess the higher level of the endocannabinoid 2-AG, which decreases in spermatocytes (~2-fold) and in spermatids (~20-fold), while AEA is constantly present during spermatogenesis. Accordingly, spermatogonia express higher and lower levels of 2-AG biosynthetic and degrading enzymes, respectively, as compared to meiotic and postmeiotic cells (not shown). Scale bar: 10 µM. ECS—Endocannabinoid system; CB—Cannabinoid receptor; 2-AG—2-arachidonylglycerol; AEA—Anandamide.

Mammalian male germ cells, from mitotic to haploid stage, have a complete and active ECS which is modulated during spermatogenesis [12,51]. In the mouse, ECS has been hypothesized to control at least two critical steps of the germ cell differentiation required for the success of spermatogenesis as indicated in Figure 1. At an early phase of spermatogenesis, testicular 2-AG, produced at a high level by spermatogonia, might act as an autocrine factor via CB_2_, promoting their meiotic entry. In the final steps of spermatogenesis, AEA, produced by Sertoli cells [52] and/or by spermatids [12], might act on spermatids as a paracrine/autocrine factor, via CB_1_, regulating spermiogenesis (Figure 1). In this way, continuous sperm production, throughout the reproductive lifetime, is guaranteed by the action of ECS that controls the balance between meiotic entry of spermatogonia and spermiation. A similar expression pattern of many components of the ECS has been recently described in human germ cells, suggesting that eCBs might also play a role in the regulation of human spermatogenesis [53]. Consequently, exogenous cannabinoids exposure, as in *Cannabis C. sativa* users, could over-activate the system with a negative impact on male fertility. Interestingly, in humans, *C. sativa* use, is associated with impotence, decreased testosterone level, impairment of spermatogenesis, reduction of sperm motility and viability; more recently, it has been associated with alterations in sperm epigenome in humans, rats [54], and mice [55]. This last observation is strongly relevant for the potential impact on the gamete function of transmitting information to the next generation.

## 5. Cannabinoid Receptors in Male Germ Cell Development

Cannabinoid receptors are members of the superfamily of seven-transmembrane-spanning receptors and are coupled with G proteins. Both cannabinoid receptors, CB_1_ and CB_2_, are implicated in male reproductive biology [2,35,36]. However, they seem to have specific expression in germ cells at different stages of differentiation and distinct roles in regulating fertility. Here we will discuss on the importance of cannabinoid receptors signaling in the regulation of several stages of male germ cell development and their role in mediating epigenetic modifications.

### 5.1. Cannabinoid Receptor CB_1_

CB_1_ is prominently expressed in the central nervous system (CNS) and has attracted great attention as a modulator of different brain functions. It is most abundant in the hippocampus, basal ganglia, cerebellum, and prefrontal cortex and is involved in a variety of physiological functions including appetite, fear, anxiety and pain [56,57,58]. However, it has also been detected in peripheral tissues including the reproductive system. CB_1_ is encoded by the gene *CNR1* and consists of 472 amino acids in humans, 473 amino acids in rats and mice, with 97–99% amino acid sequence identity among these species. In addition to the canonical long form, the presence of splice isoforms both in humans and mice [59], coming from 5′-UTR introns of the gene, have been described. These three isoforms are differentially expressed in the human brain, skeletal muscle, liver, and pancreatic islet [60] and via different signaling properties, contribute to the CB_1_ receptor physiology.

In the testis, CB_1_ is expressed by somatic and germ cells of mammalian and non-mammalian vertebrates and its activity is correlated to the Leydig cell differentiation, steroidogenesis, spermiogenesis, sperm maturation, and quality. In both rat and mouse, a key role for CB_1_ has been demonstrated in Leydig cell development, and its expression in these cells positively correlates with differentiation events and negatively with respect to their proliferation [47,61].

In mouse germ cells, CB_1_ mRNAs expression is detectable in fetal gonocytes starting from E11.5 and their expression level remains low and constant during embryo development and after birth [62]. A higher level of CB_1_ starts to be expressed during spermatogenesis in haploid cells and became more evident in sperm, indicating a role of this receptor in the final steps of germ cell differentiation such as spermiogenesis and acquisition of functional properties. It has been demonstrated that *Cb1−/−* male mice show inefficient histone displacement and produce spermatozoa with uncondensed chromatin and damaged DNA [63] indicating that CB_1_ is involved in spermiogenesis and, in particular, plays a role in chromatin remodeling by regulating histone displacement and Tnp2 expression levels. 

Mouse sperm express an even higher level of CB_1_, and its activation causes adverse effects on sperm function including inhibition of motility, capacitation, and acrosome reaction [64]. On the other hand, in the absence of CB_1_ signaling, sperm acquire motility precociously and the percentage of motile spermatozoa recovered from the caput of the epididymis is higher with respect to wild-type mice, suggesting a physiological role of this receptor in controlling sperm motility in the epididymis [48]. Physiologically, a gradient of the endocannabinoid 2-AG in the epididymis prevents activation of sperm motility in caput, through activation of CB_1_ [65]. Similarly, in humans, CB_1_ is expressed by sperm and its activation inhibits motility by decreasing mitochondrial activity [66], while CB_1_ inhibition through the use of rimonabant, a CB_1_ antagonist, is able to increase sperm motility and viability and to induce acrosome reaction and capacitation [67].

In human sperm, CB_1_ receptor is localized in the plasma membranes of the head and middle piece and has been also identified intracellularly on the mitochondria membrane (mtCB_1_) [66,67,68,69]. Although the expression of functional intracellular CB_1_ in mitochondria has been demonstrated [70,71] in other tissues such as brain [72] and skeletal muscles [73], where it can regulate cellular respiration and other bioenergetic processes [71,74]; the role of mtCB_1_ in sperm is not entirely clarified. The fact that mitochondria are the principal suppliers of sperm energy and that cannabinoids are potent inhibitors of sperm mitochondrial O2 consumption [69] suggests that mtCB_1_ could mediate adverse effects of cannabinoid drugs on mitochondrial functionality and thereforeexplain the negative effects on sperm motility.

In human sperm cells, CB_1_ has been found co-localized with the vanilloid receptor TRPV1, known as the heat-sensing receptor [75]. TRPV1 is activated by temperatures higher than 42 °C [76,77] and has been suggested to be a mediator of sperm thermotaxis in humans [78] and to play a role in the stabilization of the plasma membranes in capacitated sperm [79]. Mammalian spermatozoa, immediately after ejaculation, are unable to fertilize the oocytes and acquire this competence during the transit within the female genital tract. Sperm cells undergo a series of morpho-functional modifications, known as “capacitation” [80] that allow them to become able to recognize the oocyte and to extrude the content of acrosomal vesicle (acrosome reaction, AR), thus penetrating the zona pellucida (ZP) and reaching the oocyte membrane. It has been proposed that both the receptors CB_1_ and TRPV1 [81,82] could participate in the modulation of spermatozoa maturation allowing sperm to acquire fertilizing ability [82,83]. Specifically, CB_1_ could be implicated in the Gi protein/cAMP/PKA pathway in the early stages of post ejaculation, promoting the maintenance of membrane stability and avoiding premature acrosome reaction. TRPV1, on the contrary, could be activated in the latest stages of capacitation determining the rapid increase in intracellular calcium concentration needed for acrosome reaction. The observation that TRPV1 expression, at mRNA and protein level, is not limited to human sperm cells but has been detected also in murine germ cells from spermatocyte to spermatozoa and in Sertoli cells [12,78] suggests its potential protective role against heat stress and in conferring heat resistance to male germ cells [84].

### 5.2. Cannabinoid receptor CB_2_

CB_2_ is referred to as the peripheral cannabinoid receptor since it is predominantly expressed in the immune system [85] where it participates in the regulation of immune responses and in mediating the anti-inflammatory effects of *C. sativa* [86]. However, CB_2_ shows a moderate expression in other peripheral tissues, including the cardiovascular system, gastrointestinal tract, liver, adipose tissue, bone, and reproductive system. More recently a functional CB_2_, expressed in neurons of the hippocampus, has been identified [87]. CB_2_ is encoded by the gene CNR2 and consists of 360 amino acids in humans. Two isoforms of the CB_2_ have been identified in humans: hCB_2_A and hCB_2_B. Strikingly, these two isoforms show a tissue-specific expression: hCB_2_A is mainly expressed in the testis, more than 100-fold than in spleen and leukocytes, whereas the other hCB_2_B is expressed predominantly in spleen and at lower level in other peripheral tissues except the testis [88]. The expression of the testis-specific isoform might indicate that hCB_2_A could regulate functions related to spermatogenesis and fertilization. However, detailed information on the expression and role of hCB_2_A in human testis to date are unknown. Agirregoitia et al. reported the expression of CB_2_ in human sperm and suggested that, along with CB_1_, it could be also involved in sperm motility regulation [89]. However, various evidence indicates that CB_2_ is expressed at a higher level in germ cells at early stage of differentiation in mice, rats, and humans [12,51,53]. It is already expressed by gonocytes in fetal mouse testis starting from E11.5 and its expression increases during embryo development reaching a very high level in spermatogonia at birth [12]. In postnatal mouse testis, CB_2_ continues to be expressed by spermatogonia and its expression dramatically decreases in spermatocytes, reaching a very low level in spermatids and disappearing in mouse spermatozoa. Interestingly, spermatogonia possess also the higher level of the endocannabinoid 2-AG, which decreases in spermatocytes (~2-fold) and in spermatids (~20-fold; see Figure 1). Accordingly, spermatogonia express higher and lower levels of 2-AG biosynthetic and degrading enzymes, respectively, as compared to meiotic and postmeiotic cells. Altogether these observations indicate the involvement of an autocrine/paracrine endocannabinoid signaling mediated by CB_2_ receptor and sustained by 2-AG, which may regulate several functions in mitotic male germ cells. In this context, it has been demonstrated that activation of CB_2_, through the use of the selective agonist JWH-133, promoted *in vitro* meiotic entry of mouse spermatogonia [12] while it did not affect mitotic germ cell proliferation (P.G., unpublished observation). Morphological and molecular evidence supported these conclusions, since CB_2_ activation in spermatogonia increased: (a) the number of SYCP3 positive cells, corresponding to early meiotic prophase stages, (b) the expression of early meiotic genes, and (c) the expression of the meiosis-specific histone H3K4me3 methyltransferase Prdm9. PRDM9 trimethylates specific H3K4 sites, at meiotic entry, specifying the recombination hotspots, essential for progression through prophase I [30]. Accordingly CB_2_ activation in spermatogonia increases the global level H3K4me3 and induced histone modifications at promoter regions of meiotic and premeiotic genes *c-Kit* and *Stra8*, compatible with their transcriptional activation. All these events occur physiologically during spermatogenesis when B-type spermatogonia enter meiosis and reach the leptotene stage of prophase I, suggesting that CB_2_ could play a physiological pro–meiotic role in spermatogenesis, controlling the timely coordinated progression of spermatogenesis. Notably, chronic administration of JWH-133 to immature male mice induces an acceleration of the onset of spermatogenesis, whereas the specific CB_2_ antagonist delays germ cell differentiation, thus demonstrating that both hyper- and hypo-stimulation of CB_2_ disrupted the temporal dynamics of the spermatogenic cycles [13]. These findings highlight the importance of proper CB_2_ signaling in the testis for the maintenance of a correct temporal progression of spermatogenesis. Disruption of the temporal dynamics of the spermatogenic cycle has important clinical implications because it frequently leads to reduced fertility or infertility due to increased germ cell apoptosis [90]. Regarding CB_2_, very recently, we have demonstrated that the hyperactivation of this cannabinoid receptor in male mice, besides promoting germ cell differentiation, reduced sperm number recovered by cauda epididymis [55]. This apparent discrepancy could be explained by a loss of the accelerated germ cells caused by apoptosis. Accordingly, a similar effect has been demonstrated in fetal oocyte at meiotic entry. In females, activation of CB_2_ signaling in fetal oocytes exerts a pro-meiotic effect *in vitro* and causes, *in vivo*, an increase in apoptotic cell death that leads to reduced ovarian reserve at birth [62].

### 5.3. Role of Cannabinoid Receptors in Epigenetic Modifications during Male Germ Cell Development

Recent evidence in humans and animal models reported that activation of cannabinoid receptors, through the exposure to cannabinoids, is associated with epigenetic modifications [91]. Indeed, *in vitro* and *in vivo* experiments have reported that cannabinoid treatment induces alterations in DNA methylation and histone modifications in several cell types. In human keratinocytes, it has been demonstrated that cannabinoids regulate the expression of skin differentiation genes through DNA methylation [92,93], while Rotter et al. reported that CB_1_ expression is regulated by DNA methylation in peripheral blood cells in subjects with THC dependence [94]. Along the same line, another study addressed THC-induced epigenetic changes in immune cells showing histone modifications in some genes of lymph node cells in mice [95]. Regarding the CNS, it is known that the brain is particularly vulnerable to cannabinoid exposure, which can lead to adverse effects resulting in mental health disorders. In a study in which the molecular basis for this brain vulnerability was investigated, the authors identified histone modifications in three rat brain areas (hippocampus, nucleus accumbens, and amygdala), after adolescent and adult chronic THC exposure [96]. Similarly, Tomasiewicz et al. reported an increased Penk gene expression in response to rat adolescent THC exposure associated to changes in histone methylation [97].

The effect of cannabinoids on epigenetics has been also investigated during prenatal exposure in the developing fetus, via maternal exposure during pregnancy. A study on the immune system in mice showed that in utero exposure to THC resulted in markedly defective T cell differentiation and impaired T cell function in offspring. This immunosuppressive effect has been correlated to epigenetic mechanisms such as altered microRNA, DNA methylation, and histone modification profiles [98]. In another study, maternal cannabis use has been reported to alter the developmental regulation of mesolimbic dopamine D2 receptors in offspring through histone lysine methylation [99]. A summary of studies reporting associations between post-natal (A)/prenatal (B) exposure to cannabinoids and epigenetic alterations is shown in Table 1.

**Figure 2:**
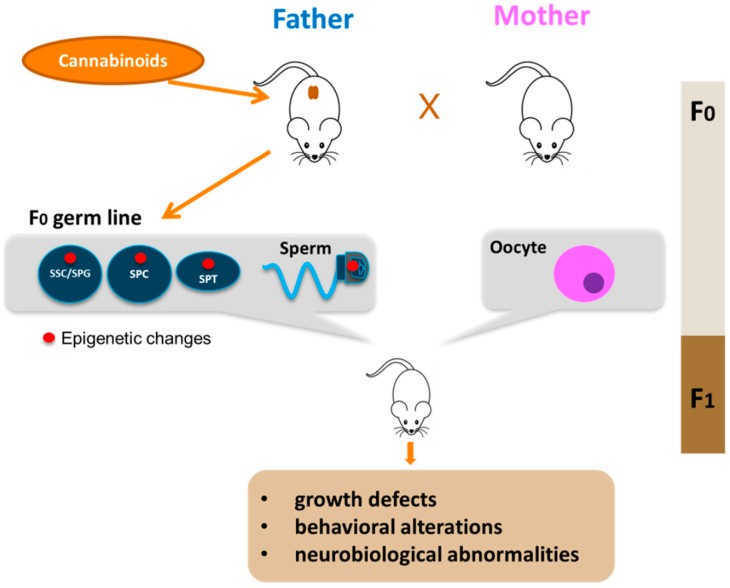
Paternal transmission of cannabinoid-induced epigenetic modifications. Cannabinoid exposure, particularly that during young age, leads to epigenetic alterations in the germline of the father (red circle). The epigenetic aberrations could appear in spermatogonial stem cells (SSC) or in spermatogonia (SPG) and could be maintained during germ cell differentiation in meiotic cells (SPC), haploid cells (SPT) up to sperm. Epigenetic alterations are then transmitted to F1 offspring by sperm with consequences on offspring health.

Differently from the somatic cell types, epigenetic modifications in the germline are especially important because they can be transmitted to the progeny. Although compelling evidence is now showing that father exposure to cannabis can induce heritable changes in the sperm epigenome, very few studies have up to now addressed this point. In *in vitro* experiments on isolated mouse male germ cells, we reported alteration of H3K4me3 and H3K9me2 levels at the promoters of *c-Kit*, *Stra8* and *Gfra1* genes in mouse spermatogonia treated with the CB_2_ agonist JWH-133 [13], underlining the susceptibility of these cells to epigenetic modifications. A very interesting study of Murphy et al. showed that cannabis use in humans, and THC exposure in rats, is associated with widespread changes in sperm DNA methylation [54]. From this study, they identified hypomethylation in autism candidate gene DLGAP2 in the sperm of human and rat exposed to *C. sativa.* Moreover, they found the same hypomethylated state in this gene in the nucleus accumbens of rats born from THC-exposed fathers [105], strongly supporting the potential for intergenerational inheritance of altered sperm DNA methylation patterns. Some other studies are beginning to shed light on cannabis/cannabinoid-induced epigenetic modifications paternally transmitted. Szutorisz et al. reported that THC exposure of male and female adolescent rats resulted in behavioral and neurobiological abnormalities in the subsequent F1 generation as a consequence of parental germline exposure to the drug [106] and, in a different report, they showed that these defects were associated to altered gene expression in the nucleus accumbens due to modified DNA methylation [107]. Levin et al. reported that paternal THC exposure in rats induced DNA methylation alterations in sperm and this correlated to impairment in attentional performance in the offspring [108], while, another study showed that male exposure to cannabinoids during adolescence induced stress vulnerability in the offspring and this effect was associated to increased global DNA methylation in the offspring prefrontal cortex [109]. All these studies reveal that paternal exposure to cannabis and cannabinoids is associated with various behavioural and neurobiological abnormalities in the offspring through epigenetic mechanisms transmitted by sperm cells. Very recently, we investigated the effects of paternal selective activation of CB_2_ on offspring. We found that chronic exposure of prepubertal male mice to CB_2_ agonist JWH-133 induced sperm DNA hypermethylation at paternally expressed imprinted genes *Plagl1* and *Peg10*, important for placental development and offspring growth. The hypermethylation level in these imprinted genes correlated to decreased expression of Tet genes. Interestingly, these specific alterations in sperm epigenome were inherited by the embryonic tissues and caused defects in placental and embryonic growth [55]. Overall, these studies clearly demonstrated that paternal cannabinoid receptors overactivation can induce epigenetic alterations in male gametes that are then transmitted to the next generation with an impact on offspring health as indicated in Figure 2. A summary of studies reporting associations between parental exposure to cannabinoids before conception and epigenetic alterations transmitted to the progeny is shown in Table 2. Altogether these evidence underline the susceptibility of male germ cells to epigenetic modifications following drug exposure and highlight the critical role of sperm as key vector of inheritance.

## 6. Cannabinoid Receptors and Testicular Tumours

The role of the endocannabinoid system in the pathophysiology of cancer is not completely clear yet. Most studies point out that in humans CB receptors are upregulated in tumours and that their overexpression correlates with cancer [110]. However, the activation of CB receptors in human cells in vitro and in mice models has often an anti-tumorigenic effect, acting on cancer cell proliferation, migration, apoptosis, and tumor vascularization. The mechanisms are complex and can differ between cancer types. In addition, a selected number of tumours might be targeted by the inactivation instead of stimulation of cannabinoid receptors. This highlights the complexity of the endocannabinoid system and the need for a careful molecular characterization of the tumours and patients stratification before agonists or antagonists of cannabinoid receptors might be employed, alongside with standard treatments, to enhance therapy efficacy. 

Testicular tumors are the most frequent solid tumors of adolescents and young adult males. Indeed they can appear at any age with three apparent subtypes of distinct molecular etiology: prepubertal testicular tumors, present in boys younger than 12 years of age, post-pubertal testicular tumors comprising the adult testicular tumors present in young men between 15 and 40 years of age, and spermatocytic tumours that generally are present in men older than 50 years of age [111]. By far, the most prevalent subtype is postpubertal adult testicular tumors, with a lifetime risk of about 0.5–1%, that in some countries have increased up to three-fold in the last five decades [112,113,114,115]. In 2016, the WHO classification of testicular tumors was revised, considering advances in the understanding of their tumorigenesis and molecular features. These changes led to a division into two major groups: prepubertal-type tumors, not derived from germ cell neoplasia in situ (GCNIS), and postpubertal-type tumors, GCNIS-derived [116]. The GCNIS-derived tumors are much more common and typically occur in postpubertal men from 18 to 45 years of age. The histotypes include seminoma, embryonal carcinoma, yolk sac tumor, trophoblastic tumors, teratoma, mixed germ cell tumor, and regressed germ cell tumor. The prepubertal-type germ cell tumors, the other major category of testicular tumors not derived from GCNIS, occur mostly but not exclusively in children. Spermatocytic seminoma has been designated as a spermatocytic tumor and placed within the group of non–GCNIS-related tumors. Postpubertal testicular tumors are believed to originate from a common precursor, the GCNIS cell [116,117]. GCNIS cells stay quiescent during infancy, followed by proliferation in puberty, probably due to hormonal stimulation, with subsequent progression into overt tumors. An essential feature of GCNIS is its location at the base of seminiferous tubules, in the “spermatogonial niche” and the expression of markers such as OCT3/4, placental alkaline phosphatase, and AP-2ɣ. The biological causes of the initial malignant transformation from a precursor cell to a GCNIS cell are still unclear. The initial transformation most likely takes place *in utero* during the early development of the germline and the target cells are most likely the embryonic germ cells, either PGCs or gonocytes [118,119,120,121].

The incidence of testicular cancer varies by ethnic origin, with the highest rates reported in developed countries and lowest in developing countries. Several risk factors have been identified for testicular tumors. Beyond age, race, and family history of the disorder, a strong risk factor is cryptorchidism (undescended testis) [122]. Other factors include infertility [123] and genetic conditions such as Down’s syndrome [124], or XY gonadal dysgenesis [125], suggesting that alterations of germ cell development, inherited factors, or congenital genetic changes play a role in the pathogenesis of testicular tumors. Although the activation of CB receptors has been associated with an anti-cancer effect in a variety of human cells and mice models, recently, marijuana use has also been evaluated as a risk factor for testicular cancer development by increasing the incidence of testicular tumors [126,127,128,129,130,131]. Interestingly, the reported association of *C. sativa* exposure and increased incidence of testicular tumors concerns exclusively non-seminomas. However, the molecular mechanisms behind this association are currently unknown. It is thus likely that active compounds in phytocannabinoids, by binding to cannabinoid receptors at a central level, could target the hypothalamic–testis axis, thus disrupting normal hormone regulation of spermatogenesis and leading to carcinogenesis. Moreover, cannabinoids could bind, at the periphery in the testis, to CB receptors expressed in germ cells or somatic cells, altering germ cell development and firing the cancerous transformation.

Furthermore, the lack of GCNIS or seminoma-like tumors in rodents (and in particular of mice models) impairs the performance of molecular studies which might help a better understanding of these human diseases. Further studies are warranted in the years to come, to furthermore elucidate the mechanism.

Although a large body of literature has explored the role of CB receptors in a variety of tumors, only recently, a single study described a pro-apoptotic effect of the cannabinoid receptor agonist WIN 55,212-2 on a testicular cancer cell line [132].

**Figure 3:**
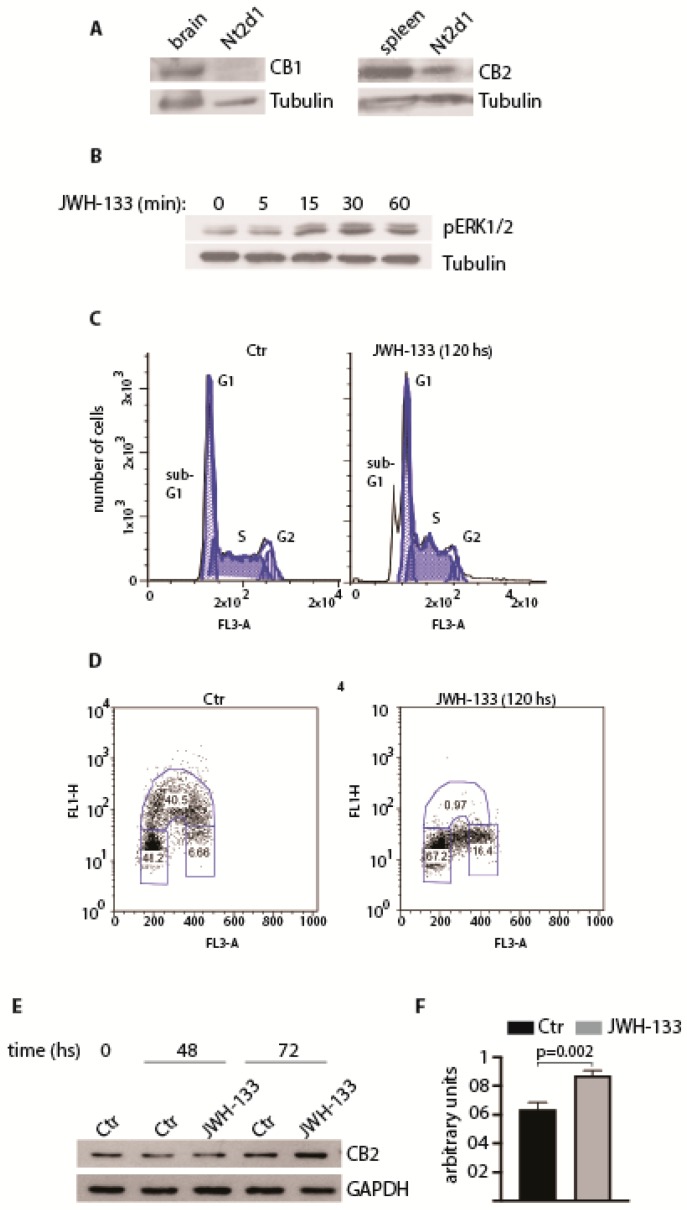
Cannabinoid receptor CB_2_ in embryonal carcinoma. (**A**) Expression of CB_1_ and CB_2_ receptors in Nt2d1 cell line. Mouse brain and spleen were used as positive controls for the expression of CB_1_ and CB_2_, respectively. (**B**) Analysis of the activation of ERK pathway following the stimulation with 1 µM JWH-133 at the indicated time points. In (A) and (B), tubulin was used as loading control. (**C**) Cell cycle profile of Nt2d1 cells in untreated and JWH-133 treated cells (1 µM), at the indicated time point. The sub-G1 pick is indicative of nuclear fragmentation. (**D**) Chronic exposure of Nt2d1 cells at 1 µM JWH-133 causes an arrest at the G1/S phase of the cell cycle. (**E**) Expression of CB_2_ receptor following chronic exposure of Nt2d1 cells at 1 µM JWH-133 for the indicated time frames. The expression of glyceraldehyde 3-phosphate dehydrogenase (GAPDH) was used as loading control. (**F**) Densitometric analysis of CB_2_ expression treatment of Nt2d1 cells with 1 µM JWH-133 for 72hs. CB_2_ expression was normalized against the loading control (GAPDH). Data are mean value ± s.d. of three independent experiments. Statistical analysis was performed using a unpaired two-tail Student’s *t*-test (*p* < 0.05; Barchi and Grimaldi, unpublished data).

With the goal of characterizing CB receptors expression and their potential role in testicular tumours, we describe below our preliminary unpublished findings on the embryonal carcinoma cell line NTERA-2 cL.D1 (Nt2d1) [133], a pluripotent testicular tumour cell line that is extremely sensitive to cisplatin treatment due to its low proficiency in the repair of cisplatin-induced damage [133] and to P53-mediated apoptosis (see [134] and references therein). By Western blotting analysis, we found that Nt2d1cells expressed CB_2_ but not CB_1_ receptors (Figure 3A). To test whether CB_2_ receptor was functional, we treated Nt2d1 cells with the CB_2_-selective agonist JWH-133, using a dose that we observed to be biologically active in germ cells [12]. We observed that CB_2_ receptor activation induced transient ERK1/ERK2 phosphorylation (Figure 3B) and cell death by apoptosis, as demonstrated by the presence of a peak in the sub-G1 stage by analysis of the cell cycle profiles (FACS; Figure 3C). Moreover, the analyses of cell proliferation, measured by evaluating the incorporation of the thymidine-analog bromodeoxyuridine/5-bromo-2′-deoxyuridine (BrdU), revealed that chronic treatment with JWH-133 caused a strong reduction of BrdU incorporation (Figure 3D), indicating an arrest of the cell cycle at G1/S transition. In some cellular contexts, prolonged treatment with CB_2_ agonists can increase the expression of the receptor, leading to an increased proficiency in downstream signaling activation [110]. To understand whether this molecular mechanism could account for the anti-proliferative effect of JWH-133 in Nt2d1 cells, we analyzed CB_2_ protein level at different time points after stimulation with JWH-133. As shown in Figure 3E and quantified in Figure 3F, CB_2_ expression was significantly up-regulated, suggesting that the response of Nt2d1 cells to the drug is correlated to the sustained expression of CB_2_ level and signaling. Overall our results indicate that CB_2_ activation might have a therapeutic potential for the treatment of testicular tumours. Further studies will be required to reveal the effect of CB_2_ activation in cisplatin-resistant cells in vitro and in vivo, alone or in combination with other therapeutic drugs [135].

## 7. Concluding Remarks

The cannabinoid receptors are expressed in male germ cells at any stage of their differentiation and their downstream signaling is involved in the physiological progression of germ cell development and in sperm functions. The presence of a complete and active ECS in these cells suggests that they are also sensitive to exogenous cannabinoids. Marijuana, or *C. sativa* is the most widely used illicit drug and its use is strongly increasing in the last years, potentially as a result of the widespread legalization for both medical and recreational use. It is also the drug most commonly used by young people at reproductive age. The fact that *C. sativa* exerts its biological effect through activation of cannabinoid receptors prompt a strong need to understand the impact of cannabinoid exposure on germ cells. More recently, the interest is increased following the identification of epigenetic implications of cannabis exposure in germ cells. Indeed these cells are unique in their ability to transfer genetic/epigenetic information from generation to generation. As such, the integrity of their genome and epigenome are paramount to the health of organisms of the next generation. On this basis, recent findings have begun to highlight that fathering cannabinoid exposure induced alterations in the sperm epigenome and these alterations were associated with increased defects in the offspring, including behavioural and neurobiological abnormalities and reduced growth at birth and fetal life. Moreover, considering that testicular tumours are believed to arise from failure of normal maturation of gonocytes, the risk of a potential predisposition in the offspring to TGCT or to metabolic and chronic diseases in adulthood should be considered in future studies.

## Figures and Tables

**Table 1:** Epigenetic changes associated to cannabinoids exposure.

**1.A. Epigenetic Changes that Occur within the Lifespan Due to Direct Cannabinoids Exposure.**				
**Drug**	**Biological Target**	**Epigenetic Marks**	**Associated Effects**	**Reference**
THC	Peripheral blood cells (human)	CB_1_ and CB_2_ promoter methylation	Decreased CB1 expression in blood cells	[94]
THC	Immune cells (mouse)	Histone modifications: - H3K4me3 - H3K9me3; - H3K27me3; - H3K36me3; - H3K9ac	Pleiotropic effect on gene expression in immune cells	[95]
THC	- Hippocampus - Nucleus accumbens - Amygdala (rat)	Histone modifications: - H3K9me2,3 - H3K27me3 - H3K9ac - H3K14ac	Vulnerability to psychiatric disorders	[100]
THC	Adult brain (rat)	Histone modifications (H3K4me3; H3K9me3)	Increased *Penk* gene mRNA levels	[97]
THC	Mouse myeloid-derived suppressor cells	miRNAs	Altered miRNA involved in myeloid expansion and differentiation	[101]
THC	Intestine (macaque)	miRNAs	Induction of anti-inflammatory microRNA expression	[102]
WIN55,212-2	Adult mouse brain (hippocampus)	DNA methylation	Decreased expression of *Rgs7*; memory impairment	[103]
**1.B. Epigenetic changes that occur during fetal life due to direct in utero cannabinoids exposure.**				
**Drug**	**Biological Target**	**Epigenetic Modification**	**Associated Effects**	**Reference**
THC	Adult nucleus accumbens (rat)	Histone modification (H3K4me3; H3K9me2)	Decreased *Drd2* gene expression level	[99]
THC	Human trophoblast cell line (BeWo)	Increased HDAC3 expression	Gene dysregulation during placental development	[104]

THC—Δ9-tetrahydrocannabinol; WIN—WIN55,212-2 synthetic cannabinoid; CB—Cannabinoid receptor; H3K—lysin of histone 3; HDAC—Histone deacetylase; Rgs7—Regulator of G-protein signaling 7 gene; Drd2—Dopamine receptor D2 gene; Penk—Proenkephalin gene.

**Table 2:** Epigenetic changes that occur in parental germline before conception and transmitted to the F1 generation.

Drug	Biological Target	Epigenetic Modification	Associated Effects	Reference
JWH-133	Spermatogonia (mouse, in vitro)	Histone modification (H3K4me3; H3K9me2)	Accelerated entry into meiosis	[13]
THC/Cannabis	Sperm (rat/human)	global DNA methylation	Altered hippo signaling and cancer pathways in sperm	[54]
Cannabis	Sperm (rat/human)	DNA methylation	Hypomethylation in autism DLGAP2 gene in sperm and nucleus accumbens of offspring	[105]
THC	Adult nucleus accumbens (rat)	DNA methylation	Altered methylation in genes associated with neurotransmission and synaptic plasticity genes in F1 offspring	[107]
THC	Sperm (rat)	DNA methylation	Impairment in attentional performance in offspring	[108]
WIN55,212-2	Sperm (rat)	DNA methylation	Increased DNA methylation in offspring prefrontal cortex associated with stress vulnerability	[109]
JWH-133	Sperm (mouse)	DNA methylation	Hypermethylation at imprinted *Peg10* and *Plagl1* genes in sperm and placenta. Altered placental and embryonic growth	[55]

JWH—JWH-133 synthetic CB_2_ agonist; DLGAP2—Disks large-associated protein 2 gene;Peg10-Paternally expressed gene 10; Plagl1—PLAG1 Like Zinc Finger 1.
